# Spontaneous network activity <35 ​Hz accounts for variability in stimulus-induced gamma responses

**DOI:** 10.1016/j.neuroimage.2019.116374

**Published:** 2020-02-15

**Authors:** Jan Hirschmann, Sylvain Baillet, Mark Woolrich, Alfons Schnitzler, Diego Vidaurre, Esther Florin

**Affiliations:** a Institute of Clinical Neuroscience and Medical Psychology, Medical Faculty, Heinrich Heine University, 40225, Düsseldorf, Germany; b McConnell Brain Imaging Centre, Montreal Neurological Institute, McGill University, Montréal, QC, H3A2B4, Canada; c Wellcome Centre for Integrative Neuroimaging, Department of Psychiatry, Oxford Centre for Human Brain Activity (OHBA), University of Oxford, Oxford, OX3 7JX, United Kingdom; d Wellcome Centre for Integrative Neuroimaging, Nuffield Department of Clinical Neurosciences, Oxford Centre for Functional MRI of the Brain (FMRIB), University of Oxford, Oxford, OX3 9DU, United Kingdom; e Center for Movement Disorders and Neuromodulation, Department of Neurology, Medical Faculty, Heinrich Heine University, 40225, Düsseldorf, Germany

## Abstract

Gamma activity is thought to serve several cognitive processes, including attention and memory. Even for the simplest stimulus, the occurrence of gamma activity is highly variable, both within and between individuals. The sources of this variability, however, are largely unknown.

In this paper, we address one possible cause: the cross-frequency influence of spontaneous, whole-brain network activity on visual stimulus processing. By applying Hidden Markov modelling to MEG data, we reveal that the trial-averaged gamma response to a moving grating depends on the individual network dynamics, inferred from slower brain activity (<35 ​Hz) in the absence of stimulation (resting-state and task baseline). In addition, we demonstrate that modulations of network activity in task baseline influence the gamma response on the level of trials.

In summary, our results reveal a cross-frequency and cross-session association between gamma responses induced by visual stimulation and spontaneous network activity. These findings underline the dependency of visual stimulus processing on the individual, functional network architecture.

## Highlights

• Pre-stimulus and resting-state activity correlate with induced gamma responses.
• Hidden Markov Modelling reveals correlations on the level of subjects and trials.
• Dependencies exist across brain areas, frequency bands and recording sessions.
• The results show an influence of individual network profiles on induced responses.

## 1 Introduction

Narrow-band gamma activity can be observed in numerous species and brain areas with various recording techniques (Bosman et al., 2014), including M/EEG recordings in humans (Jensen et al., 2007). It has been proposed to play a role in a variety of cognitive processes, including attention (Bosman et al., 2012; Grothe et al., 2012), feature binding (Engel et al., 1991; Singer and Gray, 1995), memory encoding (Sederberg et al., 2003; Jutras et al., 2009), memory retrieval (Osipova et al., 2006; Montgomery and Buzsaki, 2007), decision-making (van Wingerden et al., 2010, 2014), and reward processing (Berke, 2009; Kalenscher et al., 2010).

Importantly, gamma responses to visual stimuli vary substantially within and between subjects. Invasive recordings in monkeys (Lundqvist et al., 2016) and humans (Kucewicz et al., 2014) revealed that gamma responses of the same individual vary markedly from trial to trial. In fact, single-trial gamma responses have been described as transient events of varying amplitude, duration and frequency. These findings suggest that the oscillation-like appearance of the trial-averaged gamma response might be a misleading consequence of averaging, not reflecting the actual physiological processes engaged in single trials (Jones, 2016; van Ede et al., 2018). Still, averaging across trials results in a remarkably reproducible pattern, as shown by MEG studies measuring trial-average gamma responses in human visual cortex repeatedly in the same subjects (Hoogenboom et al., 2006; Muthukumaraswamy et al., 2010). Between subjects, in contrast, the trial-average response differs markedly with respect to amplitude, frequency and bandwidth (Muthukumaraswamy et al., 2010) and this between-subject variability has been shown to have a relatively strong genetic basis (van Pelt et al., 2012).

To date, the cause of within- and between-subject variability in gamma activity is not completely understood. Here, we propose that gamma responses might differ between subjects because subjects differ in the dynamics of basic, intrinsic networks which are common to all tasks and contexts (Finn et al., 2015). According to our hypothesis, these inter-individual differences become apparent even in the absence of gamma-inducing stimuli, implying that resting-state activity can predict gamma responses. This idea is based on functional magnetic resonance imaging (fMRI) (Smith et al., 2009; Cole et al., 2014, 2016; Tavor et al., 2016) and one recent MEG study (Becker et al., 2018), which demonstrated that resting-state network activity predicts inter-individual differences in task-related brain activity. Rest-task cross-frequency relationships affecting gamma oscillations, however, have not been investigated so far.

Notwithstanding the existence of robust networks, the brain is able to adapt flexibly to changes in the environment. Hence, we propose that network dynamics do not only reflect the individual, but also, possibly to a lesser extent, the current situation. With respect to gamma activity, this assumption implies that induced responses might differ between trials because individual network activity is modulated within a task.

**Fig. 1:**
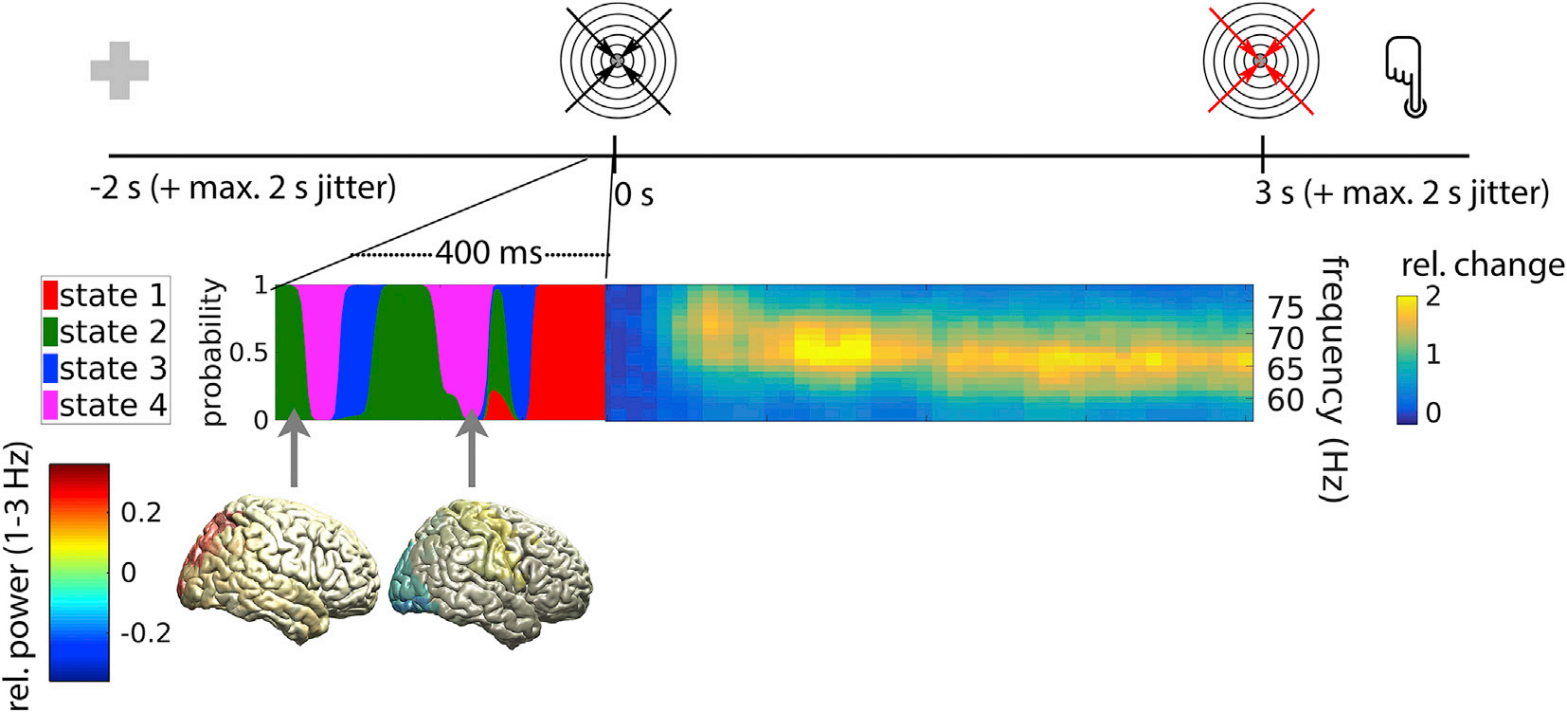
Experimental paradigm and rationale of the study. Upper row: Timeline of a single trial, locked to grating onset. Following a baseline period of 2–4 ​s with a central fixation cross, an inward-moving grating appeared which accelerated at an unpredictable moment 3–5 ​s following grating onset (illustrated here with red arrows). Subjects indicated they detected the acceleration via a button press. Lower left: Hidden Markov Modelling yielded at each time sample the probability of the brain being in any of four states (color-coded). State inference was limited to epochs without stimulation, i.e. to the pre-stimulus baseline, as shown here, or to the resting-state (not shown). Each state is characterized by a unique spatio-spectral profile within the frequency range below gamma (1–35 ​Hz), including the topography of delta power shown here for states 2 (left) and 4 (right). Lower right: The inward-moving grating induced strong gamma activity in occipital areas. We investigated whether the strength of this stimulus-induced gamma response is related to spontaneously occurring whole-brain states. (For interpretation of the references to color in this figure legend, the reader is referred to the Web version of this article.)

To test these hypotheses, we derived an estimate of network dynamics by applying Hidden Markov Modelling (HMM) to whole-brain MEG data, describing re-occurring patterns of network activity as repeated visits to a finite set of brain states (Fig. 1). Using the HMM, we investigated whether gamma responses differ between subjects because some subjects spend more time in certain brain states than others (between-subject effect). In addition, we tested whether the amplitude of the gamma response differs between trials because the pre-stimulus brain state differs between trials (within-subject effect). And finally, we compared the predictive potential of task baseline vs. resting-state activity with respect to gamma amplitude.

## 2 Methods

### 2.1 Experimental design

#### 2.1.1 Participants

15 healthy participants were recruited for this study (21–45 years; 5 female). The study was approved by the Montreal Neurological Institute’s ethics committee (NEU 011–036) and was in accordance with the Declaration of Helsinki. All participants gave written informed consent and were compensated for their participation.

#### 2.1.2 Paradigm

Subjects were presented with a modified version of the visual stimulation paradigm by Hoogenboom et al. (2006): An inward-moving, circular sine wave grating with a diameter of 5° accelerated from 1.6 deg/s to 2.2 deg/s at an unpredictable moment between 3 and 5 ​s after stimulus onset. Subjects indicated that they had detected the velocity change by pressing a button with the index finger of the dominant hand. The button press ended the trial and the stimulus was turned off. During the inter-trial interval (baseline period), subjects were presented with a central fixation cross. Inter-trial intervals varied between 2 and 4s. A few trials with longer interval (17–19 ​s) were randomly interspersed in the trial sequence for all subjects but P1 (6–16 per subject; mean: 13). This was done to facilitate an analysis of the influence of baseline duration, but is not relevant for the analyses reported here.

#### 2.1.3 Experimental procedure

Each session started with a 5 ​min resting-state recording with eyes open, which was immediately followed by task practice and task recording. Before the start of the reaction time task, participants completed 10 practice trials. The task was divided into 2–5 blocks, containing 35–78 trials each (mean: 62.85). After each block, participants received a feedback on the accuracy of their responses and had the possibility to take a break. Following the reaction time task, a further 5 ​min resting-state recording was acquired.

In two subjects (P3 and S006R), additional task data were acquired 6 days and 1 day after the first recording session, respectively. Subject P1 was not recorded in resting state.

### 2.2 Data acquisition

Participants were measured in a seated position with a 275-channel VSM/CTF MEG system at a sampling rate of 2400 ​Hz (no high-pass filter, 660 ​Hz anti-aliasing low-pass filter). Electrocardiography (ECG) and vertical electrooculography (EOG) were recorded simultaneously using MEG-compatible electrodes. Magnetic shielding was provided by a magnetically-shielded room with full 3-layer passive shielding. Participant preparation consisted of affixing 3 head-positioning coils to the nasion and both pre-auricular points. The position of the coils relative to the participant’s head was measured using a 3-D digitizer system (Polhemus Isotrack, Colchester, USA).

A T1-weighted MRI of the brain (1.5 ​T, 240 ​× ​240 ​mm field of view, 1 ​mm isotropic, sagittal orientation) was obtained from each participant either at least one month before or immediately after the session.

### 2.3 Preprocessing

Data were preprocessed and analysed using the HMM-MAR (Vidaurre et al., 2016) and Fieldtrip (Oostenveld et al., 2011) toolboxes for Matlab (The Mathworks). All data were screened visually. Noisy channels and noisy epochs were excluded from analysis. Data were down-sampled to 250 ​Hz. A 60 ​Hz discrete Fourier transform filter was applied to remove line noise. Cardiac and eye movement artefacts were isolated by FASTICA (Hyvarinen, 1999) and removed in non-automatic component selection.

### 2.4 Source reconstruction

Individual T1-weighted MR scans were aligned to the MEG’s coordinate system, segmented and used for the construction of a single-shell, realistic head model (Nolte, 2003). To define a set of source coordinates, the “colin27” template MRI (Holmes et al., 1998) was inflated using FreeSurfer (Fischl et al., 1999) and a cortical mesh consisting of 2052 sources was constructed using MNE (Gramfort et al., 2014). The corresponding coordinates in individual head space were obtained by applying the inverse of the normalizing transform matching the individual to the template MR scan. The lead field (forward model) was computed based on the source coordinates and the head model. Subsequently, a Linearly Constrained Minimum Variance (LCMV) spatial filter (Van Veen and Buckley, 1988) was computed based on the lead field and the sensor covariance matrix, and data were projected through this filter trial-by-trial.

To reduce dimensionality, we grouped sources into parcels, defined by the Talairach Tournoux atlas (Lancaster et al., 1997), and carried out all subsequent analyses on the parcel level. First, each source was either assigned to one of 25 bilateral brain areas of interest or discarded from further analysis if it was more than 5 ​mm away from an area of interest (325 of 2052 sources). The areas of interest consisted of all cortical areas contained in the atlas, with the exception of seven areas at the base of the brain or deep within the interhemispheric fissure, which were assumed to have poor MEG signal quality (rectal gyrus, parahippocampal gyrus, subcallosal gyrus, transverse temporal gyrus, orbital gyrus, and uncus). The 25 bilateral brain areas of interest were further sub-divided into a left- and a right-hemispheric parcel, resulting in 50 cortical parcels of interest (Tab. 1 of the Supplementary Material). The first principle component was extracted from each parcel of interest and magnetic field spread between parcels was reduced by symmetric, multivariate orthogonalization (Colclough et al., 2015).

Note that the sign of this processed beamformer output is arbitrary. Because our analysis requires sign consistency across subjects, we applied a sign flipping procedure to maximize sign consistency; see (Vidaurre et al., 2016) and (Vidaurre et al., 2018b) for details.

### 2.5 Stimulus-induced gamma activity

We quantified post-stimulus gamma responses in order to relate them to brain states inferred from slower activity (≤35 ​Hz) occurring in the pre-stimulus baseline period or the resting-state recordings. Post-stimulus gamma responses were computed by multitaper spectral estimation using 2 Slepian tapers (Thomson, 1982). Power was estimated for frequencies between 40 ​Hz and 100 ​Hz in a 300 ​ms sliding segment which was moved in steps of 50 ​ms. At each time step, the segment was Fourier-transformed, multiplied with each of the two, Fourier-transformed tapers, and the products were averaged over tapers. We screened post-stimulus parcel activity and identified a frequency band, a time window and a location of interest. Because individual gamma peak frequencies varied markedly across subjects (between 42 and 74 ​Hz), frequency selection was subject-specific, i.e. we defined an individual gamma band for each subject (individual gamma peak frequency ±10 ​Hz). The time window of interest was set to 0.6–2 ​s relative to stimulus onset because all subjects were found to exhibit stable gamma activity in this window. The bilateral cunei were chosen as the locations of interest because this was generally the area with the strongest gamma response. For the analyses described in the following, gamma power within ±10 ​Hz of individual gamma peak frequency was normalized by computing, for each frequency and each trial, the percent change relative to mean power in the response baseline (−0.5 to −0.2 ​ms from grating onset). Subsequently, gamma power was averaged over frequency, time and locations of interest.

### 2.6 Hidden Markov models

HMMs are probabilistic sequence models that find recurring patterns in time series data (Rabiner and Juang, 1986). Unlike sliding-window approaches, they can reveal fast state changes present in multichannel, electrophysiological recordings (Baker et al., 2014; Vidaurre et al., 2016, 2018a, 2018b). HMMs describe the dynamics of brain activity as a sequence of transient events, each of which corresponds to a visit to a particular brain state. For each state, the HMM infers a time-course that describes the probability of that state being active. Furthermore, each state is characterized by a unique spatio-spectral profile. In summary, HMM brain states can be considered a compact description of multi-faceted, recurring patterns in dynamic network activity. HMMs have been widely used in a variety of applications, such as the decoding of speech (Varga and Moore, 1990), the comparison of nucleotide sequences (Eddy, 1998) or the detection of pathological brain signals (Hirschmann et al., 2017; Kottaram et al., 2019). They are used here to categorize multi-channel data in an unsupervised fashion, similar to network analyses based on Independent Component Analysis (Brookes et al., 2011) or *k*-means clustering (Cabral et al., 2017). The HMM is, however, more flexible and data-driven in including the different spectral properties within the data e.g. phase-coupling (Vidaurre et al., 2018b), without relying on previous filtering or computations of power envelopes.

### 2.7 State inference

States were inferred separately from the baseline periods of the task and the resting-state recordings. For the baseline period, the first second of each trial was removed because it was assumed to contain activity related to the button press of the previous trial. Next, we *z*-scored and concatenated the data from all subjects in time, resulting in a total of 71.44 ​min of pre-task rest data (per subject mean: 5.10 ​min, STD: 1.40 ​min), 164.85 ​min of baseline data (per subject mean: 10.99 ​min, STD: 2.19 ​min) and 63.47 ​min of post-task rest data (per subject mean: 4.53 ​min, STD: 0.69 ​min). The rest recording preceding the task and the rest recording following the task were combined by concatenation in the time dimension to increase the amount of resting-state data available for state inference. Importantly, we applied a spectral filter with a pass-band of 1–35 ​Hz to ensure that brain states were not based on gamma activity. This was done to demonstrate the universality of rest-task/baseline-task interactions, which we hypothesized to occur across frequency bands.

State inference was performed by applying a variety of the HMM designed to capture transient patterns of power and phase-coupling, referred to as Time-delay Embedded HMM (TDE-HMM; Vidaurre et al., 2018b). In this model, each state is characterized by certain patterns of cross-correlation, which contain spectrally-defined patterns of power and phase-coupling. The TDE-HMM parameters were chosen as in (Vidaurre et al., 2018b).

Similar to the frequency resolution in spectral analysis, the number of states *K* in a HMM determines the level of detail of the solution. Here, we set *K* ​= ​4 to guarantee a reasonable amount of trials per state, and to provide enough detail to investigate the question at hand. Similar results were obtained for *K* ​= ​3.

### 2.8 State properties

Following state inference, we computed the power and coherence associated with each state as detailed in (Vidaurre et al., 2016). In short, we used a Fourier-based multitaper approach to obtain a time-frequency representation of the concatenated data with high temporal resolution. The resulting time series of Fourier coefficients was weighted by the state probabilities, i.e. multitaper Fourier transformation, defined as

$$S\left(f\right)=\frac{1}{\sqrt{R}}\overset{R}{\underset{r=1}{\sum }}\overset{T}{\underset{t=1}{\sum }}\delta_{t}^{\left(r\right)}y_{t}e^{-2\pi ift}$$

was modified as follows:

$$S^{\left(k\right)}\left(f\right)=\frac{1}{\sqrt{R}}\overset{R}{\underset{r=1}{\sum }}\overset{T}{\underset{t=1}{\sum }}\rho_{t}^{\left(k\right)}\delta_{t}^{\left(r\right)}y_{t}e^{-2\pi ift}$$

Here, *R* denotes the number of tapers, *T* the number of time points, *δ* the taper, *y* the signal, *f* the frequency, and *t* the time point. *ρ* denotes the state- and time-specific weight, defined as the posterior probability of a state *k* at time *t* relative to its mean over time:

$$\rho_{t}^{\left(k\right)}=\frac{\sqrt{\gamma_{t}^{\left(k\right)}}}{\sqrt{\sum_{t=1}^{T}\gamma_{t}^{\left(k\right)}/T}}$$

Note that frequency measures such as power and coherence are theoretically defined for each time point (Huang et al., 2009). Therefore, as discussed in (Vidaurre et al., 2018b), estimation of very low frequencies within states is possible even though state visits are short, insofar as there is enough total time in the time series for that state.

As a result of spectral estimation, we obtained a multi-region pattern of power and coherence per state. For topographic illustrations, power and coherence were interpolated on a 3D-reconstruction of the template brain after computing the relative difference with respect to the mean over states. In case of coherence, we display the average coupling with all other parcels.

State dynamics can be summarized by statistics such as fractional occupancy (FO), lifetime and interval time (See Baker et al., 2014 for formal definitions). FO quantifies the fraction of samples assigned to a given state. Here, a sample was assigned to a state if that state had the highest posterior probability at the time point in question (posterior decoding). Lifetime quantifies the duration of a state visit. Interval time quantifies the time in between subsequent visits of the same state.

## 3 Matching states across recordings

In order to match brain states occurring at rest and brain states occurring in task baseline, we correlated the spatial maps of spectral power for each pair of states using Pearson’s correlation coefficient. Pearson’s coefficient was chosen here because we wanted to find linear relations between power maps. Correlation was computed for each frequency band separately (delta: 1–3 ​Hz, theta: 4–7 ​Hz, alpha: 8–12 ​Hz, beta: 13–30 ​Hz, and gamma: 60–90 ​Hz) and the resulting correlation matrices were averaged across bands. Subsequently, we applied hierarchical clustering to the mean correlation matrix to group similar states (Johnson, 1967). This was achieved by Matlab’s *linkage* function, using the Euclidian distance as metric and the ‘ward’ method (inner squared distance) for computing the distance between clusters.

### 3.1 Statistical analyses

#### 3.1.1 Analysis of between-subject variability

We assessed whether the amplitude of trial-averaged, stimulus-induced gamma responses is related to state preferences in the baseline periods of the task and/or the rest recordings preceding and following the task. State probabilities were averaged across the entire baseline/rest recording and tested for a linear correlation with the amplitude of the trial-averaged gamma response using a non-parametric permutation test, where we use the Spearman’s correlation coefficient as the base statistic (Nichols and Holmes, 2002).

To probe the importance of regional oscillations for the between-subject effects, we also correlated regional, band-limited power with the induced gamma response across subjects. For the computation of power, baseline/resting-state data were cut into segments of 1 ​s length without overlap, Fourier-transformed and multiplied with a single, Fourier-transformed Hanning taper. Power was averaged over tapered segments and related to the amplitude of the gamma response using Spearman’s correlation.

#### 3.1.2 Analysis of within-subject variability

We hypothesized that the strength of stimulus-induced gamma activity depends on the brain state immediately before stimulus presentation (Fig. 1). To test this, we first computed, for each state, the average state probability in the pre-stimulus time window of interest, which served as state-specific trial weight. The pre-stimulus time window of interest was defined as −106 to 0 ​ms because 106 ​ms was the average state lifetime in task baseline (Supplementary Material).

Following weight specification, we computed a weighted trial average for each state using the obtained weights. This procedure can be considered a weighted (soft-assigned), within-subject grouping of trials by pre-stimulus state. Next, we tested whether the resulting trial groups consistently differed in post-stimulus gamma amplitude across subjects. This was achieved by running a Friedman test, followed by Bonferroni-corrected, post-hoc Wilcoxon rank sum tests. Note that trials were grouped by pre-stimulus state, not post-stimulus gamma amplitude, i.e. any consistent difference in gamma amplitude must be due to a relationship between pre-stimulus state and post-stimulus gamma amplitude.

To probe the importance of regional oscillations for the within-subject effects, we correlated the gamma response to pre-stimulus, baseline power (−106 to 0 ​ms) in selected frequency bands. We first computed the power envelope using the Hilbert transformation. Next, we assigned trials to one of four “pseudo-states” based on binned, pre-stimulus power. A trial was assigned to pseudo-state 4, for example, if the pre-stimulus power fell within 75% and 100% of the observed values. The remaining analysis was conducted as described above.

#### 3.1.3 Comparison of effect size

We compared the between-subject and the within-subject effects of network activity on gamma responses by correlating FO with the amplitude of the gamma response. To separate between-subject from within-subject effects for both FO and gamma activation, we regressed out the average value for each subject from the single trial time courses. Spearman correlation between the subject-specific averages yielded the between-subject effects, and the correlation between the residual, trial-specific values yielded the within-subject effect. Note that trial-specific values could only be obtained for baseline, not for resting-state recordings. FO was originally represented as a 2-dimensional matrix (number of trials x number of states). To obtain a single value per trial, we applied Principal Component Analysis and kept only the first principal component.

In order to assess the variability of the different effects, we generated 1000 bootstrapped samples for each effect, using random sampling with replacement on the level of subjects to produce an empirical distribution of correlations (Rindskopf, 1997). *p*-values were obtained by computing the fraction of random samples with correlation 1 ​> ​correlation 2.

## 4 Results

### 4.1 Resting-state activity and gamma responses

In this study, we describe 1–35 ​Hz spontaneous network activity by applying an HMM to resting-state MEG recordings and to the baseline periods of a task, respectively. An HMM estimates, for each sample of multivariate data, the probability of belonging to each of *K* possible brain states.

Using this approach, we derived four brains states from the combined resting-state recordings. Whereas it is possible to describe the data using more states, four were adequate for our purposes (see Materials and Methods).

**Fig. 2:**
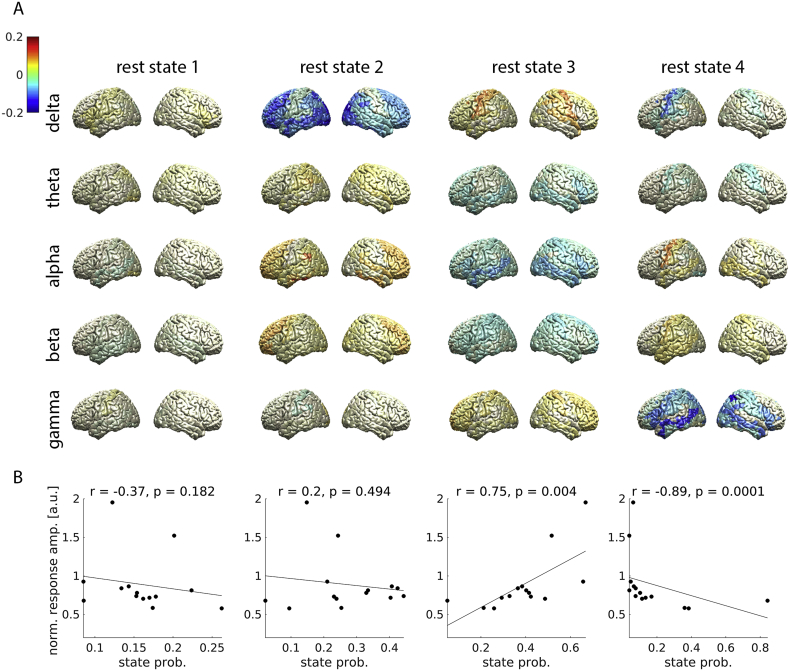
*Brain states at rest*. **A**: Power topography of each state and frequency band. Relative difference to the mean over states is color-coded. **B**: Correlation between state probability and the amplitude of the gamma response to visual stimulation. *r* ​= ​Spearman correlation. *p* ​= ​p-value. (For interpretation of the references to color in this figure legend, the reader is referred to the Web version of this article.)

We analysed the spectral properties of the brain states occurring in resting-state. Fig. 2A shows the spatial distribution of state power in the delta (1–3 ​Hz), theta (4–7 ​Hz), alpha (8–12 ​Hz), beta (13–30 ​Hz) and gamma band (60–90 ​Hz). Note that gamma oscillations did not contribute directly to state inference because they were removed by a low-pass filter, but states may differ in gamma oscillations nevertheless, e.g. through cross-frequency interactions (Jensen and Colgin, 2007). Fig. 2B shows the correlations between state probability and the amplitude of the trial-averaged, stimulus-induced gamma response. Plots on state coherence are provided in Fig. S1 of the Supplementary Material.

Rest state 3 showed a significant positive correlation with the amplitude of the trial-averaged gamma response (*r* ​= ​0.75, *p* ​= ​0.004; permutation test). This state was characterized by high delta power in central and parietal areas, low alpha and beta power in several brain regions, and high gamma power in frontal and central areas. The probability of visiting rest state 4, in contrast, correlated negatively with the gamma response (*r* ​= ​−0.89, *p* ​= ​0.0001; permutation test). This state was characterized by low delta and theta power but high alpha and beta power in central areas (Fig. 2).

### 4.2 Baseline activity and gamma responses

The results presented so far indicate that the strength of gamma responses to visual stimulation is predicted by the fraction of time spent in particular brain states occurring at rest (rest state 3 and rest state 4). We next investigated whether the same or a similar brain states occur in the baseline periods of the task. To this end, we inferred four brain states from the baseline periods of the task (see Figs. S2 and S3 of the Supplementary Material for spatial maps of power and coherence) and evaluated the spatio-spectral similarity between baseline states and the two rest states of interest by correlating spatial maps of state power and applying hierarchical clustering.

**Fig. 3:**
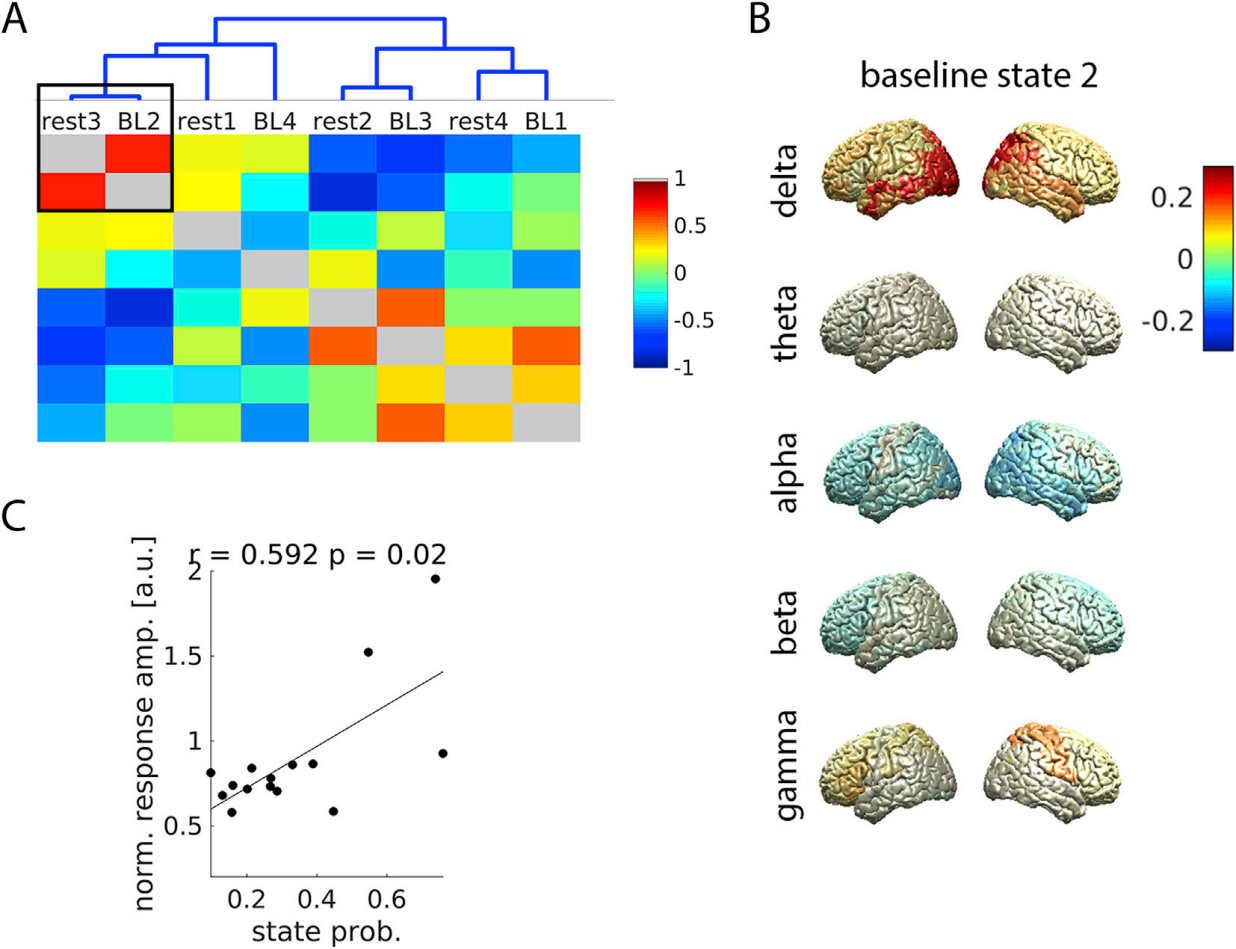
*Matching states occurring at rest and in task baseline*. **A, lower panel**: The correlation between spatial maps of spectral power for each pair of states. The Pearson correlation coefficient is color-coded. **A, upper panel**: The cluster hierarchy revealed by hierarchical clustering. The smaller the height of a bifurcation, the more similar the states. The black square marks the best match (rest state 3 and baseline state 2). **B**: Power topography of baseline state 2 for each frequency band. Relative difference to the mean over baseline states is color-coded. **C**: Correlation between the probability of visiting baseline state 2 and the amplitude of the gamma response to visual stimulation. *r* ​= ​Spearman correlation. *p* ​= ​p-value. (For interpretation of the references to color in this figure legend, the reader is referred to the Web version of this article.)

As depicted in Fig. 3A, baseline state 2 was clearly the closest match to rest state 3. Similar to rest state 3, baseline state 2 was characterized by strong delta power in parietal areas, low alpha and beta power in several brain regions, and high gamma power in central and frontal regions. A high probability of visiting baseline state 2 was associated with high amplitude gamma responses to visual stimulation (Fig.3C; *r* ​= ​0.59, *p* ​= ​0.023; permutation test). These results demonstrate that the HMM could recognize a state similar to rest state 3 in task baseline, which was likewise indicative of strong gamma responses.

A brain state clearly matching rest state 4 was not found in the baseline periods of task. The most similar state was baseline state 1, which did not correlate with the amplitude of gamma responses (*r* ​= ​−0.19, *p* ​= ​0.506; permutation test).

**Fig. 4:**
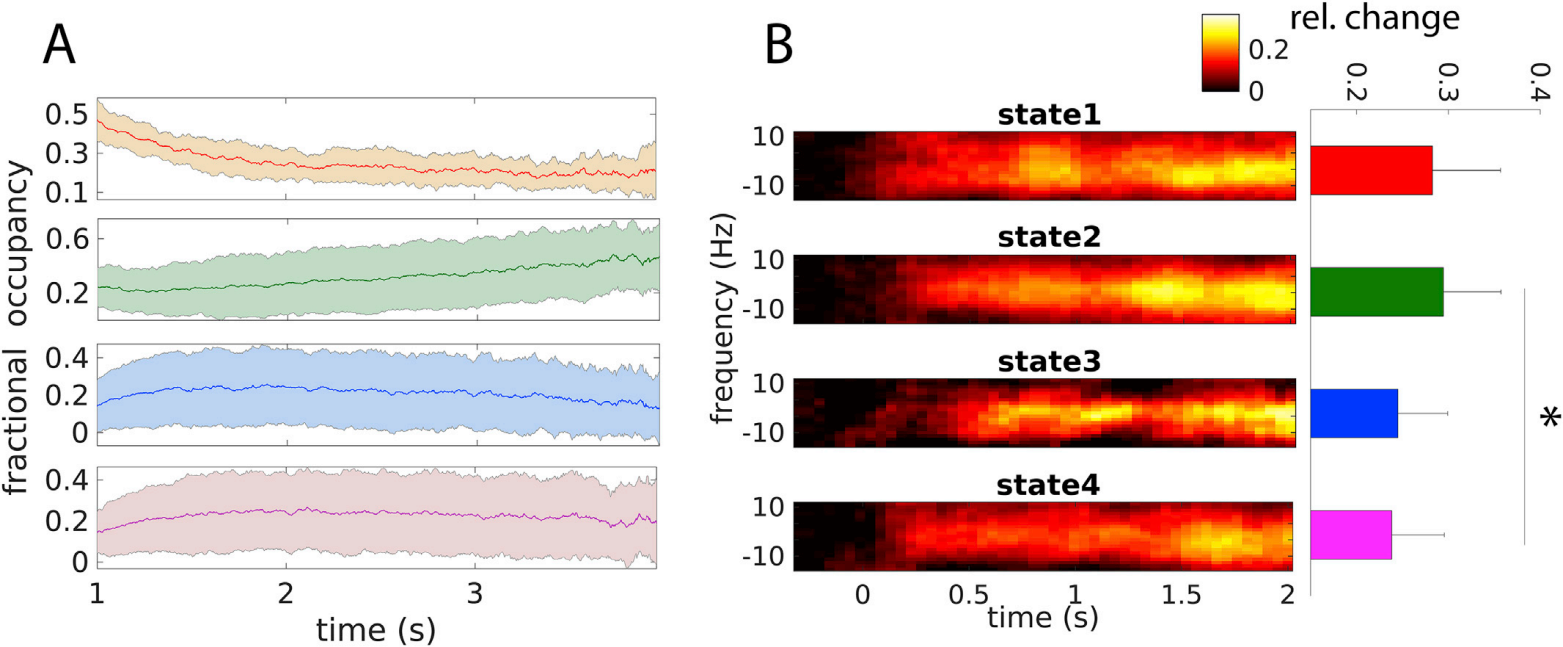
*Relationship between gamma responses and pre-stimulus brain state*. (A) Fractional occupancy in the baseline period (1–4 ​s before grating onset), averaged over subjects and time-locked to the onset of the fixation cross. Shaded areas indicate the standard deviation over subjects. The first second of each trial was discarded to reduce the effect of movement-related processing occurring after the button press. (B) Left: Weighted average time-frequency representations of gamma responses in the cuneus (left and right averaged), time-locked to the appearance of the moving grating. 0 Hz marks individual gamma-peak frequency (between 42 and 74 ​Hz). Power was baseline-corrected (−0.5 to −0.2 ​s from grating onset). Right: Power averaged over frequency (individual gamma peak frequency ±10 ​Hz) and time (0.6–2s). * corrected p ​< ​0.05.

Next, we assessed the relationship between baseline brain states and gamma responses on the level of trials (within-subject analysis). More specifically, we tested whether the baseline state occurring immediately before stimulus onset affects the amplitude of the gamma response. As illustrated in Fig. 4, gamma responses depended on the preceding baseline state (Friedman test; *p* ​= ​0.014). They were strongest when baseline state 2, i.e. the state positively correlated with the trial-average gamma response between subjects (see above), preceded grating onset. Post-hoc, pairwise testing revealed a difference between baseline states 2 and 4 (Wilcoxon rank-sum test; *p* ​= ​0.004). This pattern was observed in most individual subjects (Fig. S4 of the Supplementary Material) and was not an artefact of the trial-weighting procedure used in the analysis (Supplementary Material). In another control analysis, we verified that incompletely removed pre-stimulus eye blinks, which might impact the subsequent gamma response, were not the cause of pre-stimulus state changes (Fig. S5 of the Supplementary Material).

Due to the association with a strong gamma response both within and between subjects, we refer to baseline state 2, which likely corresponds to rest state 3, as the “gamma-enhancing brain state” in the following.

We investigated whether the gamma-enhancing brain state was upregulated as the baseline period progressed, in anticipation of the stimulus. To this end, we averaged the decoded states, time-locked to the beginning of the baseline period (fixation-cross onset), over trials. The resulting average, referred to as fractional occupancy (FO), quantifies how often a given state occurred at each time point in the baseline period (Baker et al., 2014). Indeed, baseline state 2 appeared more frequently towards the end of the baseline period (Fig. 4A; mean slope ​= ​0.086, p ​< ​0.001; *t*-test). Baseline state 1 was predominantly occurring early in the baseline, but its FO decreased over time (mean slope ​= ​−0.065, p ​< ​0.001; *t*-test). The FO of baseline state 3 showed a weak negative dependency on time (mean slope ​= ​−0.020, p ​= ​0.02; *t*-test) and baseline state 4’s FO did not change significantly (mean slope ​= ​−0.001, p ​= ​0.87; *t*-test).

Importantly, all subjects visited all brain states and none of the brain states was dominated by a small subset of subjects (Fig. S6 of the Supplementary Material), indicating that the model represented all subjects and that the HMM was able to successfully capture within-session variability.

### 4.3 Comparison of effect size

**Fig. 5:**
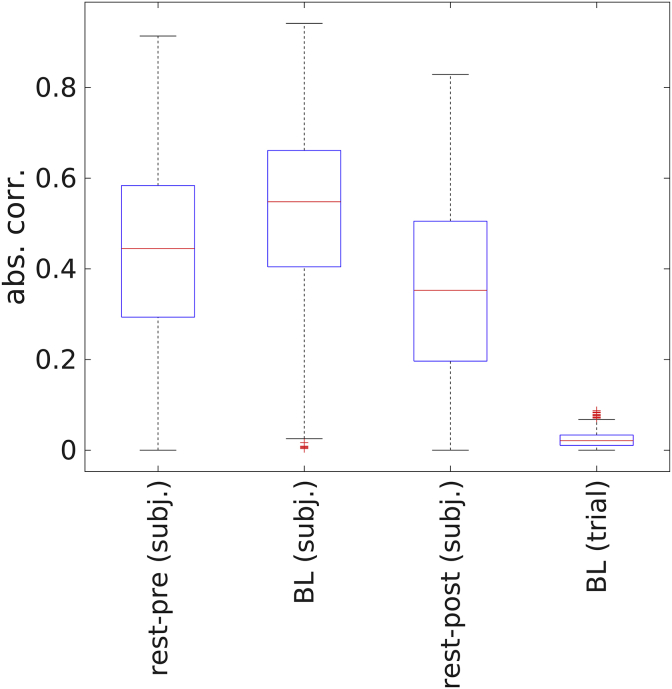
*Comparison of effect size.* Resampling subjects with replacement yielded empirical distributions of the absolute Spearman correlation coefficient. The distribution medians are represented by red lines and the 0.25–0.75 interquartile range (IR) is indicated by black whiskers. Outliers (median ​± ​2.5 IR) are represented by plus signs. *rest-pre (subj)*: between-subject correlation for resting-state recording acquired before the task; *BL (subj)*: between-subject correlation for baseline periods of the task; *rest-post (subj)*: between-subject correlation for resting-state recording acquired after the task. *BL (trial):* within-subject correlation for baseline periods of the task. (For interpretation of the references to color in this figure legend, the reader is referred to the Web version of this article.)

So far, we have revealed three different effects of spontaneously occurring brain states on stimulus-induced gamma responses: an across-subject correlation for rest, an across-subject correlation for task baseline, and a within-subject effect for task baseline. We now compare the strength of these different effects, finding a dominance of between-subject over within-subject effects. A quantitative comparison of effect size is displayed in Fig. 5. To investigate possible after-effects of task performance on resting-state activity, we considered the two resting-state recordings separately.

The within-subject effect was much weaker than any of the between-subject effects. Qualitatively, the between-subject effect was stronger for task baseline than for rest-pre and for rest-post, respectively. These differences, however, were not significant (BL vs. rest-pre: *p* ​= ​0.35, BL vs. rest-post: *p* ​= ​0.27).

### 4.4 Robustness of effects

We investigated the robustness of both the within- and the between-subjects effect in task baseline against variations in the ROI used for extracting gamma amplitude. Both effects were robust against systematic variations of response latency (Fig. S7A) and bandwidth (Fig. S7B). Changing the brain area of interest from cuneus to another area in occipital cortex, however, diminished the effect, in particular within subjects (Fig. S7C). This is most likely due to the fact that some subjects did not show clear gamma responses in the alternative areas of interest.

### 4.5 Feature analysis

Fig. 3 suggests that high delta power in central and parietal areas and low alpha power in posterior areas might be a commonality between brain states associated with strong gamma responses. Here, we tested the influence of these features directly. We first investigated the importance of brain regions by restricting the HMM to baseline activity from a centro-parietal region of interest (bilateral precentral gyrus, postcentral gyrus, paracentral lobule and superior parietal cortex) and an occipital region of interest (bilateral cuneus, inferior, middle and superior occipital gyrus), respectively. We observed a (trend) positive correlation between the trial-average amplitude of the induced gamma response and the probability of visiting one of the states in both cases (centro-parietal: *r* ​= ​0.74, *p* ​= ​0.002; occipital: *r* ​= ​0.51, *p* ​= ​0.06), similar to the between-subject association with whole-brain states. In contrast, a within-subjects effect could only be found for the centro-parietal (Friedman test; *p* ​= ​0.04) but not for the occipital model (*p* ​= ​0.56).

Next, we replaced the state probabilities by mean power in the delta (1–4 ​Hz) and the alpha band (8–12 ​Hz), respectively, to probe the importance of regional power. The gamma response correlated with mean occipital alpha power (r ​= ​−0.65, p ​= ​0.01) but not with central alpha (r ​= ​−0.42, p ​= ​0.12) or delta power (r ​= ​0.31, p ​= ​0.26) in the baseline periods of the trial.

Differences in response amplitude within subjects could not be produced by replacing state probabilities with central delta or occipital alpha power in the 106 ​ms preceding stimulus onset. We did not find differential gamma responses following different “pseudo-states” that were defined according to the level of central delta power (Friedman test; p ​= ​0.56) or occipital alpha power (p ​= ​0.65) in the pre-stimulus period of interest (−106 to 0 ​ms relative to stimulus onset).

These results indicate the between-subject relationship between brain states and gamma responses might be conveyed by local alpha power in occipital cortex, at least in the baseline period. The within-subject effect, in contrast, seems to require long-range interactions with central and parietal areas.

## 5 Discussion

In this paper, we have demonstrated that inter-individual differences in gamma responses to visual stimulation are reflected by inter-individual differences in spontaneous network dynamics. Furthermore, we have revealed a similar, albeit weaker, influence of brain states on trial-specific gamma responses. Our results imply that it is possible to predict a subject’s gamma response from their resting-state activity profile.

### 5.1 Hidden Markov Modelling of brain activity

The HMM has several useful properties for network-level analysis of electrophysiological data. Unlike sliding-window approaches, it processes the data sample by sample, facilitating the characterization of electrophysiological networks at very high temporal resolution. In addition, the HMM is a multivariate approach that considers all signals simultaneously. Rather than defining a region of interest, one can process all brain areas at once. Subsequent statistical tests do not need to be corrected for testing many areas, improving statistical efficiency. Importantly, the HMM is not a biophysical model able to explain how brain activity arises mechanistically, even though it is capable of sampling new data. Rather, it is a data-driven approach providing a compact representation of multi-channel/multi-area data.

Recent MEG studies made use of these properties to reveal a rich repertoire of fast-changing network states characterized by distinct topographies of spectral power and coupling, many of which were reminiscent of the resting-state networks originally obtained with fMRI (Baker et al., 2014; Vidaurre et al., 2016, 2018a, 2018b). HMMs and other whole-brain models have also been applied to describe dynamic connectivity in fMRI data (Cabral et al., 2017; Vidaurre et al., 2017). Here, we have used this approach to assess the relationship between spontaneous network activity (resting-state and task baseline) and stimulus-induced gamma responses.

Brain states found by an HMM can be described in different ways. Here, we focused primarily on spectral power. The feature analysis revealed that occipital alpha power might mediate the between-subject effect of brain states on the amplitude of gamma responses. The within-subject effect, in contrast, was not based on local activity in visual cortex, but might arise through interactions of visual cortex with central and parietal areas. These findings are an example of how multivariate techniques like the HMM can inform subsequent region-of-interest analyses.

We note, however, that the HMM might have used additional/other features than occipital alpha power and central delta power to categorize the data. The fact that the selected features only partially reproduced the effects of the HMM brain states indicates that other factors played a role.

### 5.2 Interactions between spontaneous and task-related brain activity

A number of fMRI studies have previously demonstrated interactions between resting-state and task-related activity (Northoff et al., 2010). Resting-state and task-related networks were found to be highly similar across a wide variety of tasks (Smith et al., 2009; Cole et al., 2014). Furthermore, stimulus-induced patterns of activation could be predicted from resting-state activity (Cole et al., 2016; Tavor et al., 2016). These findings suggest that task-related brain activity arises by relatively minor modulations of a basic network profile, which can be considered a neural signature or fingerprint, allowing for accurate identification of individual subjects (Finn et al., 2015).

The current MEG study is one the first to show that the above concept might be transferable from fMRI to neurophysiology. The fact that the individual preference for a particular brain state correlated with the individual gamma response implies that spontaneous brain activity measured in the absence of stimulation (rest or task baseline) is predictive of brain activity induced by a visual stimulus. These findings complement studies investigating spatial and spectral aspects of functional anatomy by focusing on the temporal activity profile. In the framework applied here, inter-individual differences in brain responses are thought to arise because individuals differ in the time they spend in certain brain states, defined at the population level.

So far, there is only one comparable piece of work from Becker et al., who likewise combined MEG and HMMs to predict electrophysiological responses to visual stimuli and own movements from resting-state activity (Becker et al., 2018). The current study differs from this paper in several ways. First, it investigates induced rather than evoked responses. Second, it assesses within-subject variability in addition to between-subject variability. Third, it predicts gamma band responses from low-frequency activity (cross-frequency analysis). And finally, it compares the predictive potential of resting-state and task baseline activity.

A unique insight resulting from this study is that rest-task relationships exist across frequency bands, as evidenced by an influence of spontaneous 1–35 ​Hz activity on stimulus-induced responses in the gamma band (>35 ​Hz). A possible explanation might be that fundamental brain functions like attention, which do impact brain responses and might be reflected by brain states, involve predominantly oscillations below the gamma band. This possibility is discussed in more detail below (see Brain States and Attention).

In addition, our study shows for the first time that induced gamma responses in human visual cortex are biased by pre-stimulus, spontaneous brain activity below 35 ​Hz. While this finding aligns with similar observations made for spiking (Tsodyks et al., 1999), evoked responses in local field potentials (Arieli et al., 1996; Kisley and Gerstein, 1999) and the BOLD signal (Fox et al., 2006), as well as perception (e.g. van Dijk et al., 2008; Busch et al., 2009; Baumgarten et al., 2015), it highlights one of the major advantages of our approach. The combination of MEG and HMM provides network activity resolved on a millisecond time scale, thus providing insights on the level of subjects *and* trials. On the one hand, the approach allows for estimating a subject’s average gamma response based on the brain states generally preferred by this subject (between-subject effect). On the other hand, the same model allows for estimating the gamma response in the current trial based on the brain state last visited before stimulus onset (within-subject-effect).

### 5.3 The relationship between the within- and the between-subject effect

We found a positive bias of a particular brain state both between and within subjects. Such correspondence between trial- and subject-level is not unexpected, since spending a lot of time in a gamma-enhancing state in general increases the probability of visiting this state immediately before stimulus onset. It is, however, a good indicator that the reported correlations are not driven by outliers. While it is possible that any brain state is overrepresented in strong responders by chance, it is very unlikely that the same random confound occurs on the level of trials. Conversely, differences arising by chance within subjects are not expected to translate into a relationship across subjects.

Although the direction of the between- and of the within-subject effect was identical, we found the within-subject effect to be much weaker than the between-subject effect. One possible explanation could be that baseline activity is markedly different between but rather homogenous within subjects, making trial-level predictions a more difficult task. In addition, gamma responses are noisier on the trial-level, even when considering weighted-averages instead of single trials, making them harder to predict.

### 5.4 Possible mechanisms behind gamma modulation

We can only speculate on how brain states influence gamma responses. A candidate mechanism could be the simple addition of state- and stimulus-specific gamma activity. This, however, could only explain the bias when assuming that (i) the HMM captured differences in gamma activity in the low-pass filtered data, e.g. by detecting coupled oscillations at lower frequency, and that (ii) brain states lasted from stimulus to brain response onset (600 ​ms) in a sufficient number of trials, despite a much shorter average life time (100 ​ms).

Another, trivial mechanism could be an influence of the pre-stimulus brain state on the baseline applied to trial-specific gamma responses. One might suspect the existence of such a confounder based on the observation that gamma responses were weak when baseline state 4 preceded stimulation. This state was characterized by relatively strong gamma power in posterior areas (Fig. S2). Thus, it is conceivable that, in those trials, gamma power tended to be high in the response baseline, leading to a weak baseline-corrected gamma response (the response baseline did not coincide with the pre-stimulus epoch used for state inference, but was close). If this were the case, however, one would expect medium responses following state 1, a weak response following state 2 and a strong response following state 3. This hypothetical pattern does not match the observed one (Fig. 4), suggesting that baseline effects are not confounding the interpretation given here. Further, the between-subject effect is at odds with a baseline confound: strong average gamma responses were observed in those subjects preferring rest state 3 and baseline state 2. These states were not characterized by low gamma power (Fig. 2, Fig. 3).

### 5.5 Comparing the predictive potential of baseline and resting-state activity

We investigated whether task baseline activity is more predictive of brain responses than resting-state activity. The first thing to note is that both kinds of recordings contained a similar pattern related to gamma responses (“the gamma-enhancing brain state”), indicating that prediction depends on how well the individual preference for this pattern can be estimated from a given recording. While a recent fMRI study suggests that task recordings might allow for a better discrimination between individuals than resting-state recordings (Greene et al., 2018), we observed only qualitative differences when predicting gamma responses.

It is theoretically possible that the brain states correlating positively with the gamma response represent different situations in each experimental condition, despite the spatio-spectral similarities described here. This would imply that the HMM isolated similar situations in each experimental condition which accidently share the property of enhancing gamma responses.

### 5.6 Brain states and attention

The current study did not attempt to quantify attention, and thus it cannot establish a direct link between attention and brain states. Nevertheless, there are several observations indicating that spontaneous switching between brain states in part reflects the dynamic modulation of attention. First, attention can enhance gamma responses in visual cortex, similar to the gamma-enhancing brain state observed here (Tallon-Baudry et al., 2005). Second, the gamma-enhancing brain state became more common towards the end of the baseline period, which might reflect an anticipatory upregulation of attention as stimulus presentation approached. Third, the gamma-enhancing brain state is characterized by relatively low alpha power, which is believed to reflect the current level of attention. This view is grounded in M/EEG studies showing that briefly presented visual stimuli are more likely to be perceived if posterior alpha oscillations are desynchronized (Hanslmayr et al., 2007; Dijk et al., 2008; Lange et al., 2013). When subjects are instructed to pay attention to one visual hemifield, alpha power increases in the ipsilateral hemisphere, probably to reduce the influence of distractors in the irrelevant hemifield (Mazaheri and Jensen, 2008; Treder et al., 2011; Horschig et al., 2015). Similar observations have been reported for other sensory modalities (Haegens et al., 2011; Mazaheri, 2014; Baumgarten et al., 2016), suggesting that alpha power might serve as a general mechanism for controlling whether or not a stimulus is noticed by the subject. Finally, recent MEG studies demonstrated a relationship between pre-stimulus alpha and post-stimulus gamma oscillations in visual (Popov et al., 2017) and somatosensory areas (Wittenberg et al., 2018).

Importantly, however, the gamma-enhancing brain state described here cannot be equated with low posterior alpha power. Differences in alpha power did not entirely account for the effects of brain states in this study, and low occipto-parietal alpha power was not the most prominent feature shared across states correlating positively with the gamma response. Thus, low alpha power in occipital and parietal areas might be just one aspect of sensory gating.

Assuming that brain states do reflect the level of attention, it is possible to give a rather parsimonious interpretation of our findings. First, the correlation between rest states and the trial-average gamma response indicates that it is possible to identify subjects capable of maintaining a high level of attention based on resting-state activity. The identification works equally well or even better when basing the identification on task baseline activity. This robustness against variations in recording session suggests that the individual preference for putatively attention-like brain states is not a temporary condition but rather an individual trait. It might thus be interesting to link these to genetic factors that are known to correlate with behavioural measures of attention, such as the gene for the dopamine transporter (Rueda et al., 2005) or for the neural nicotinic cholinergic receptor (Parasuraman et al., 2005). Temporary fluctuations of attention within subjects, on the other hand, might underlie the within-subjects effects reported here.

### 5.7 Limitations

While this work demonstrates a correlation between resting-state activity and brain responses, it is limited to the amplitude of induced gamma responses in the visual system. Other features such as frequency or latency were not assessed. And, unlike previous fMRI studies (Cole et al., 2016; Tavor et al., 2016) and the MEG study by Becker et al. (2018), it did not probe the predictive power of resting-state activity by generating out-of-sample predictions.

HMM state changes can in principle be caused by other factors than neuronal activity, such as noise in the data, or they could be a result of the Markovian assumption. However, there is converging evidence that relaxing that assumption does not change state life times significantly (Trujillo-Barreto et al., 2019). Also, as shown in previous work (Vidaurre et al., 2017) the HMM output can contain information at much slower time scales than the scale of state switching - an observation that is incompatible with state switching caused by noise.

## 6 Conclusions

We have shown that brains states describing spontaneous network activity <35 ​Hz are correlated with the amplitude of stimulus-induced gamma responses. Our findings suggest that each subject is characterized by individual network dynamics predictive of brain responses.
