# Supplementary material for: Spontaneous network activity <35 ​Hz accounts for variability in stimulus-induced gamma responses
Source: Neuroimage. 2020 Feb 15;207:116374. doi: 10.1016/j.neuroimage.2019.116374 (PMC8111242; doi:10.1016/j.neuroimage.2019.116374)
Supplement: Multimedia component 1 [file mmc1.doc]

# Supplementary Material

**Spontaneous network activity <35 Hz accounts for variability in stimulus-induced gamma responses**

# Topographies of power and coherence

Fig. S1 shows the spatial distribution of state coherence in the delta (1-3 Hz), theta (4-7 Hz), alpha (8-12 Hz), beta (13-30 Hz) and the gamma band (60-90 Hz) for the combined resting-state recordings. The power maps for the baseline periods are provided in Fig. S2, and the corresponding coherence maps in Fig. S3.


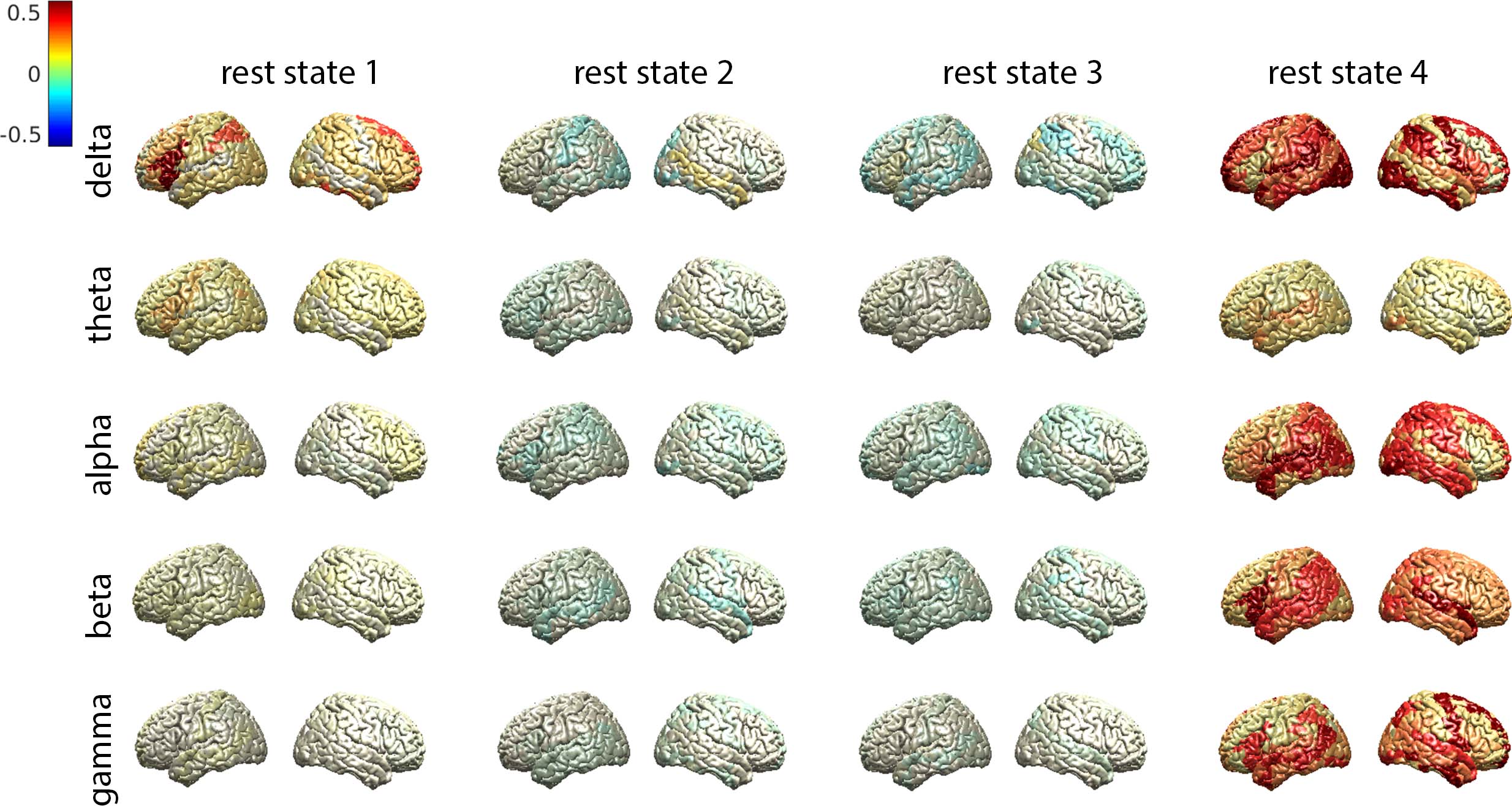


F**ig. S1:** *Topography of coherence for each rest state and frequency band.* Colours indicate the average coherence of each parcel with all other parcels, as relative difference to the mean across states.

**
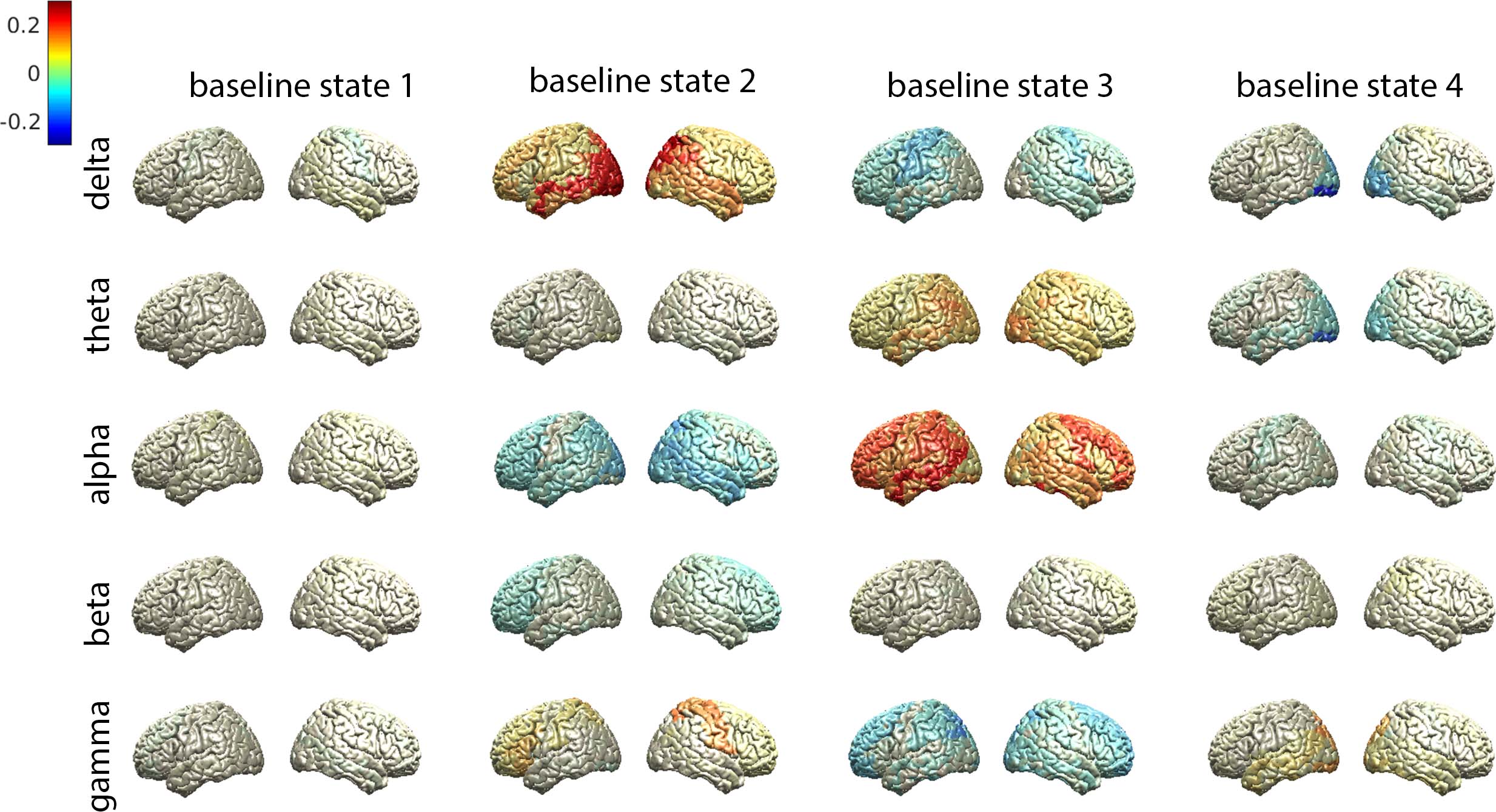
**

**Fig. S2:** *Topography of power for each baseline state and frequency band.* Colours indicate power as relative difference to the mean across states.


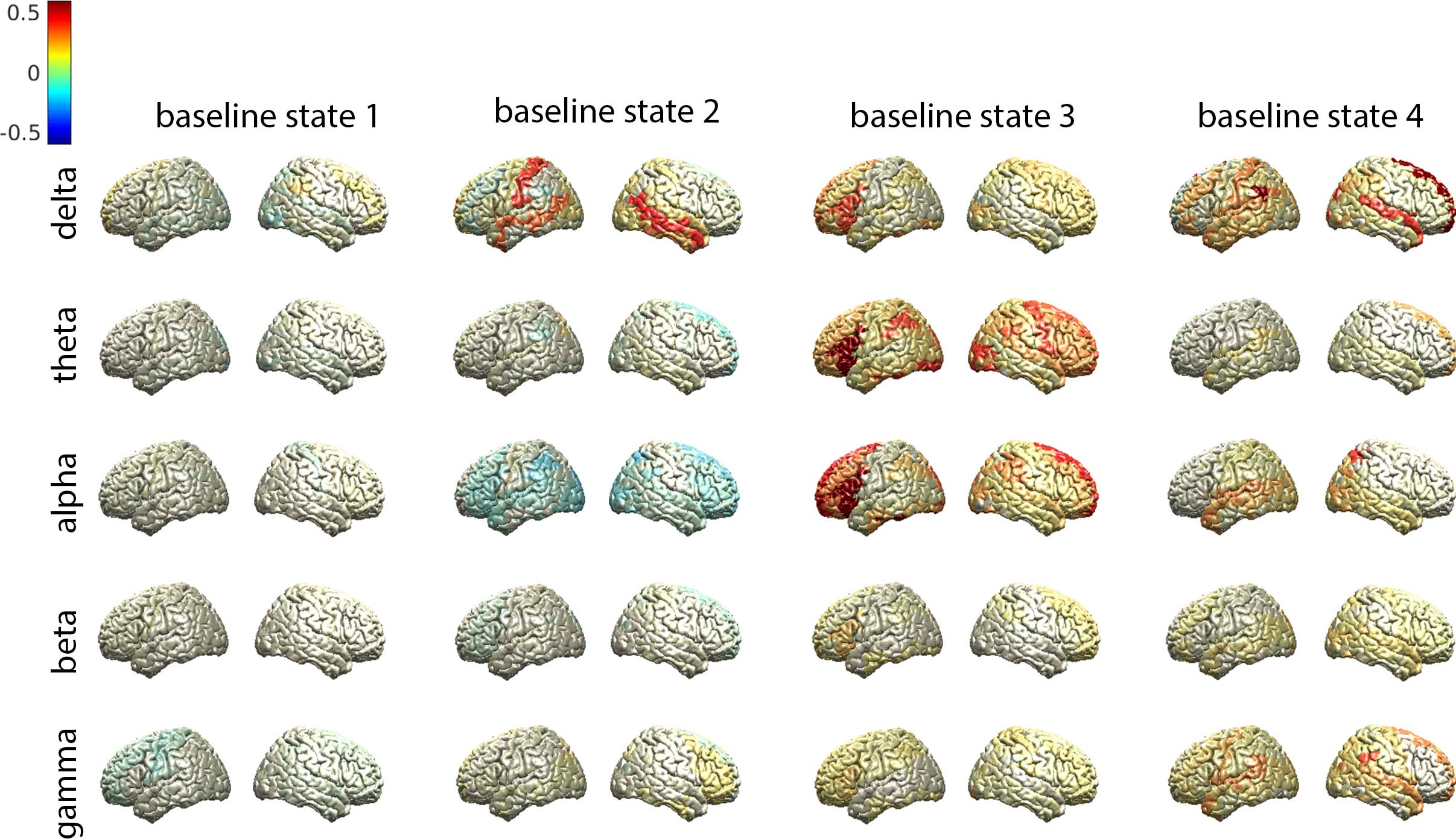


**Fig. S3:** *Topography of coherence for each baseline state and frequency band.* Colours indicate the average coherence of each parcel with all other parcels, as relative difference to the mean across states.

# Control analyses on baseline states

## Association between pre-stimulus states and induced gamma activity in individual subjects

Fig. S4 illustrates the relationship between pre-stimulus states and stimulus-induced gamma activity in individual subjects. 11 out of 15 subjects showed stronger gamma responses following baseline state 2 than following baseline state 4. The responses were approximately equal in three subjects (P1, S008 and S009) and the opposite pattern, i.e. stronger responses following baseline state 4, was observed in one subject (S007).


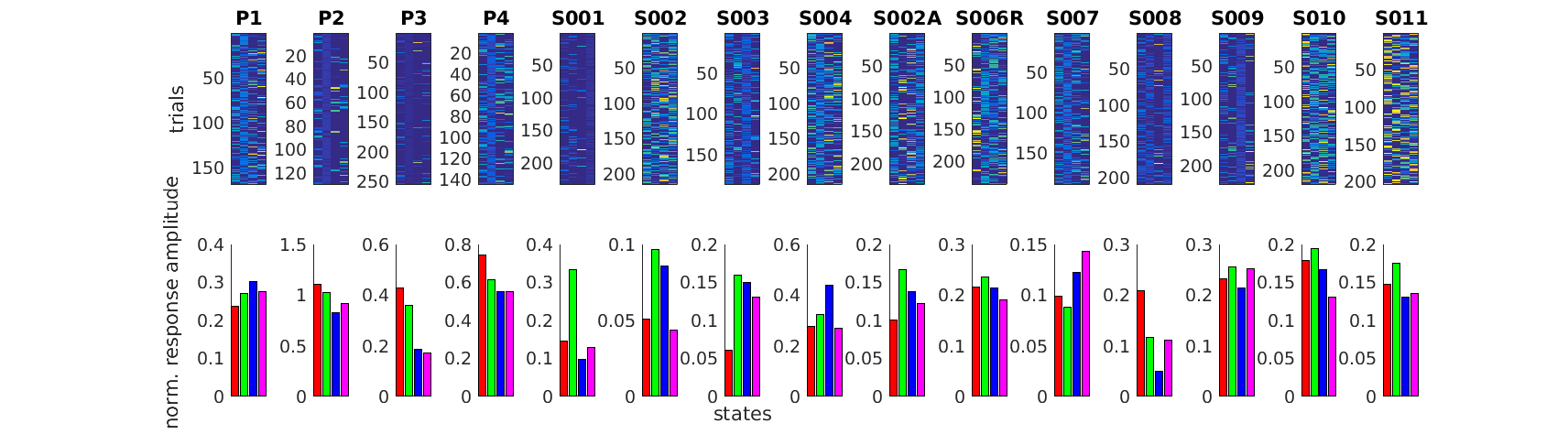


**Fig. S4**: *Relationship between pre-stimulus states and stimulus-induced gamma activity in individual subjects.* **Top:** Trial weights by state and subject (color-coded). **Bottom**: Amplitude of the stimulus-induced gamma response by preceding baseline state.

## Trial-weighting cannot explain the relationship between baseline states and stimulus-induced gamma activity

In this paper, we compared weighted averages of stimulus-induced gamma power across brain states. The trial weights were based on the state probabilities in the pre-stimulus time window of interest. This trial-weighting procedure can produce weighted average time-frequency representations (TFRs) which are similar to single-trial TFRs, i.e. noisy, if a lot of weight is assigned to individual trials. As seen in Fig. S4, upper row, considerable weight was indeed assigned to individual trials in some subjects, such as P2. If the weight concentration is systematically different between states, this can result in systematic differences between state-specific TFRs, i.e. the weight concentration might confound the interpretation. To exclude that this was the case in our data, we quantified weight concentration by information entropy and compared entropy across states. Information entropy is defined as


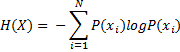


and describes the uncertainty about an outcome. It is maximal when all possible events have equal probability, i.e. when weight concentration is minimal. Entropy of baseline weights was computed for each state and each subject, and tested for consistent differences between states across subjects. Importantly, we did not find a difference in entropy (*p* = 0.26, Friedman test), suggesting that the differences between states are most likely not due the weighting procedure.

## Effects of eye blinks

To test whether baseline states depend on heartbeat or eye blinks not completely removed by ICA, we detected events in the ECG and the EOG signal, respectively, by applying individually adjusted thresholds to the high-pass filtered (>3 Hz) and z-scored ECG/EOG data. Next, we investigated whether fractional occupancy changed around these events. As depicted in Fig. S5, neither heartbeat nor eye blinks were associated with changes in fractional occupancy, implying that neither heartbeat nor blinking caused systematic state changes.

##
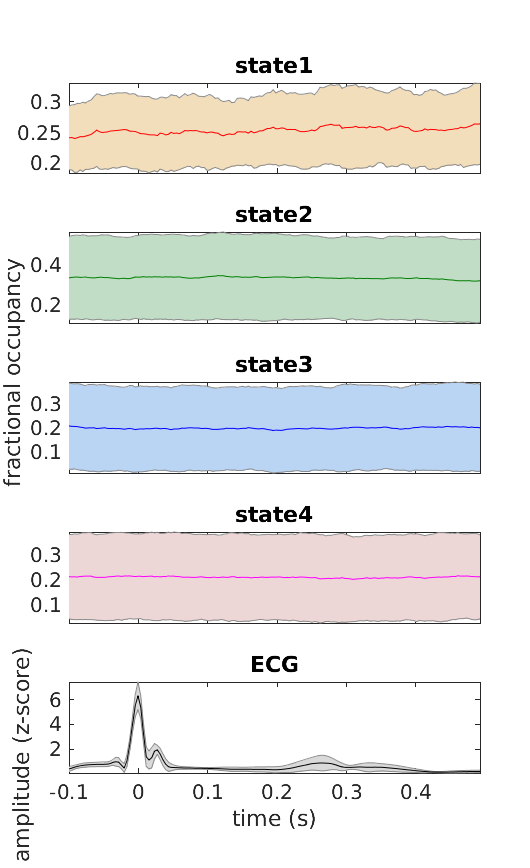

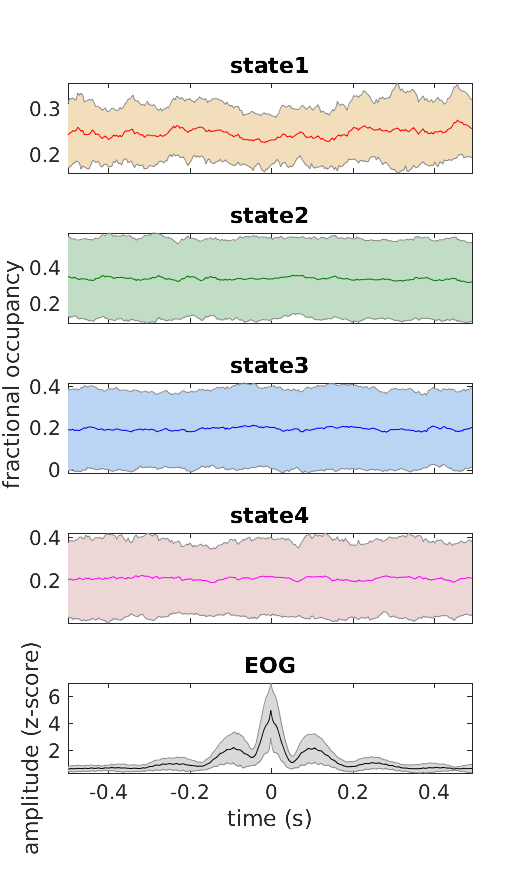


**Fig. S5**: *Fractional occupancy in relation to heartbeat (left) and eye blinks (right).* Shaded areas indicate the standard deviation over subjects. ECG = electrocardiogram, EOG = vertical electrooculogram.

## Time-domain statistics of baseline states

Fig. S6 shows fractional occupancy, mean lifetime and mean interval time for each baseline state. Fractional occupancy quantifies the fraction of samples assigned to a given state. Lifetime quantifies the duration of a state. Interval time quantifies the time in between subsequent visits of the same state. See (1) for a formal definition.

Separate one-way ANOVAs revealed no difference in fractional occupancy (F(3,56) = 0.76, p = 0.39) and no difference in mean interval time (F(3,56) = 0.32, p = 0.81) between states. There was a difference, however, in mean lifetime (F(3,56) = 6.51, p = 0.0007). Tukey-Kramer corrected post-hoc tests showed that state 1 had a longer lifetime than states 3 (mean difference = 0.07, p = 0.008) and 4 (mean difference = 0.06, p = 0.0063). The mean lifetime over baseline states was 106 ms, which defined the pre-stimulus time window of interest for detecting pre-stimulus effects (see main text, Materials and Methods).

Note that a fractional occupancy of zero did not occur for any subject (Fig. S6, upper row), indicating that all subjects visited all brain states. Although a few subjects spent up to 70% of the time in individual states, none of the states was dominated by a small subset of subjects. Such dominance would be reflected by a highly skewed distribution with most values close to zero; in this case, within-session variability would not have been captured by the HMM.


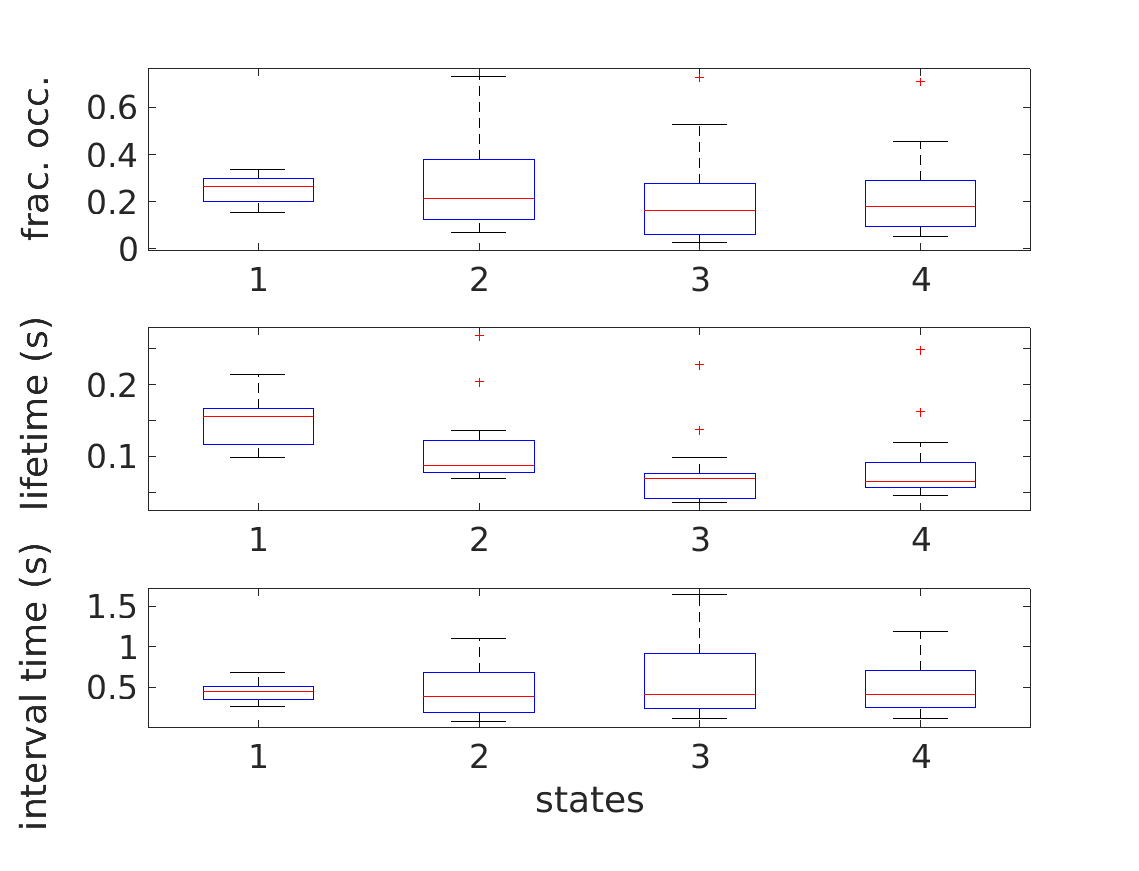


**Fig S6:** *Summary statistics of state occurrence in baseline epochs.* **Top**: Fractional occupancy. **Middle**: Mean lifetime. **Bottom**: Mean interval time.

## Robustness of state-response associations to different ROI definitions

In the main paper, we demonstrate two major influences of baseline states on gamma response amplitude: a positive correlation between the probability of visiting BL state 2 and response amplitude across subjects and a within-subject difference between gamma responses following BL state 2 and gamma responses following BL state 4. Here, we investigate whether the definition of the ROI used for the computation of gamma amplitude has any influence on these effects. Fig. S7 depicts how these effects changed when either the response latency (Fig. S7A), the band width (Fig. S7B) or the brain area of interest was varied (Fig. S7C). The original ROI was defined as follows: latency: 0.6 to 2s post-stimulus; band width: individual peak frequency ± 10 Hz; area: bilateral cuneus.

Note that none of the changes led to qualitative changes. The effect size (and significance) was comparable for different latencies and band widths, but diminished when the brain area of interest was altered. This is most likely due to the fact that some subjects did not show clear gamma responses in the alternative areas of interest.


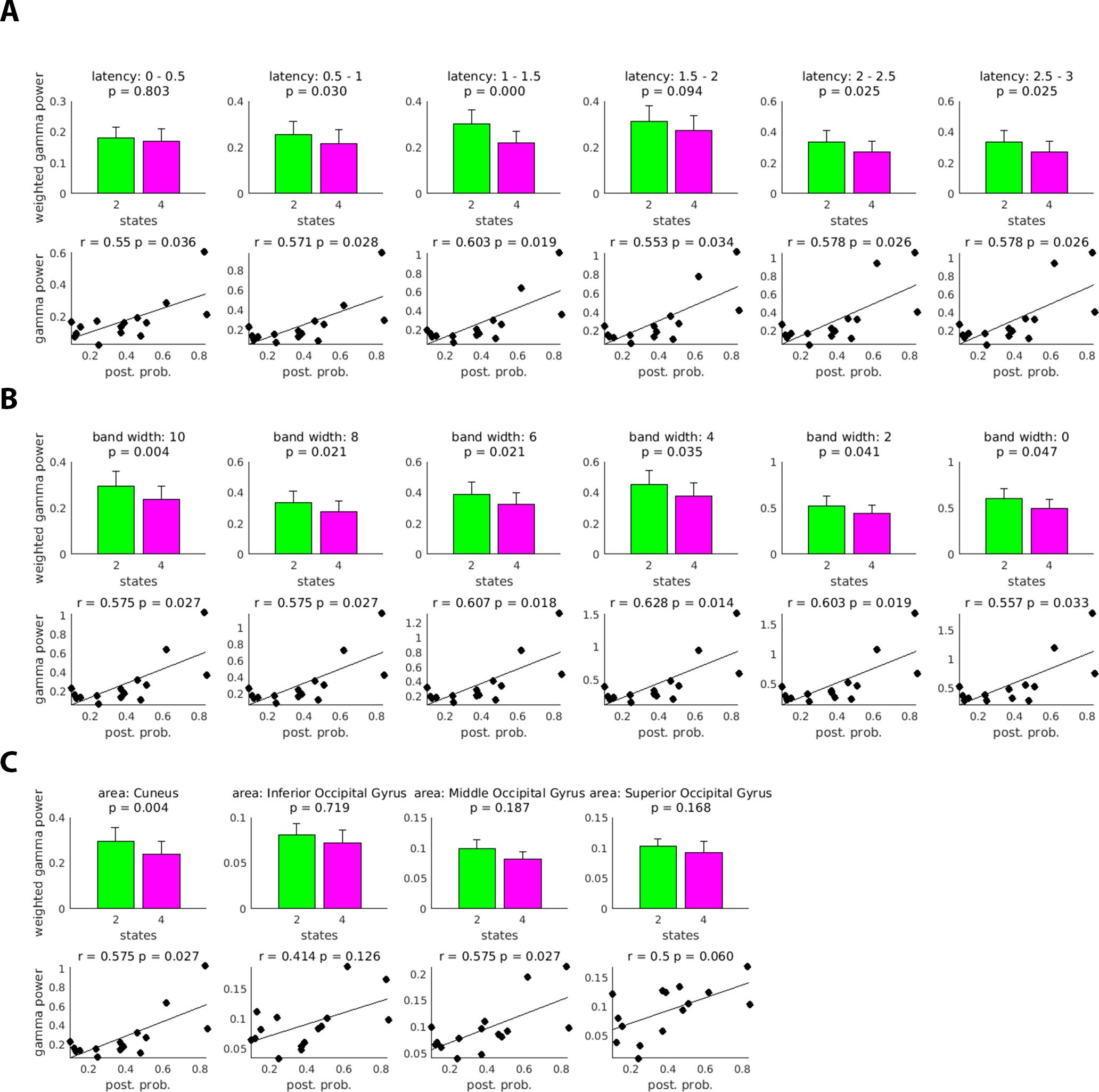


**Fig. S7: *Robustness of state-response associations to different ROI definitions.*** The upper row in each panel depicts the difference in gamma amplitude between gamma responses following BL state 2 and responses following BL state 4. The p-value denotes the result of a Wilcoxon rank-sum test. The lower row shows the Spearman correlation between the trial-average probability of being in BL state 2 and the average response amplitude. A) Variations of latency (in seconds from stimulus onset). B) Variations of gamma band width (in Hz from individual peak frequency). C) Variations of the brain area of interest.

**Tab. 1:** Parcel information. Parcel labels and Montreal Neurological Institute (MNI) coordinates of parcel centroids in millimetres. Centroid coordinates were obtained by averaging the coordinates of all sources of the cortical grid assigned to the respective parcel (see Materials and Methods). R = right; L = left.

| **parcel label** | **X** | **Y** | **Z** |
| --- | --- | --- | --- |
| Posterior Cingulate R | 11.89 | -51.50 | 15.92 |
| Posterior Cingulate L | -11.12 | -50.91 | 16.57 |
| Anterior Cingulate R | 9.23 | 26.70 | 14.12 |
| Anterior Cingulate L | -7.11 | 27.88 | 14.43 |
| Fusiform Gyrus R | 41.34 | -40.53 | -19.48 |
| Fusiform Gyrus L | -39.49 | -46.03 | -18.28 |
| Inferior Occipital Gyrus R | 36.74 | -85.05 | -9.23 |
| Inferior Occipital Gyrus L | -31.48 | -88.30 | -10.20 |
| Inferior Temporal Gyrus R | 52.10 | -21.98 | -25.09 |
| Inferior Temporal Gyrus L | -52.50 | -25.13 | -22.70 |
| Insula R | 40.92 | -12.10 | 12.67 |
| Insula L | -40.09 | -10.57 | 13.20 |
| Lingual Gyrus R | 16.75 | -73.55 | -4.56 |
| Lingual Gyrus L | -14.34 | -77.21 | -5.19 |
| Middle Occipital Gyrus R | 35.01 | -80.93 | 2.14 |
| Middle Occipital Gyrus L | -33.48 | -82.78 | 3.49 |
| Middle Temporal Gyrus R | 50.89 | -36.44 | -3.08 |
| Middle Temporal Gyrus L | -50.23 | -36.44 | -3.38 |
| Superior Temporal Gyrus R | 49.58 | -18.29 | -1.18 |
| Superior Temporal Gyrus L | -48.92 | -17.38 | -0.49 |
| Superior Occipital Gyrus R | 33.76 | -82.05 | 25.75 |
| Superior Occipital Gyrus L | -30.79 | -82.75 | 25.87 |
| Inferior Frontal Gyrus R | 42.97 | 23.20 | 4.31 |
| Inferior Frontal Gyrus L | -43.59 | 22.67 | 8.12 |
| Cuneus R | 15.80 | -86.33 | 17.68 |
| Cuneus L | -11.50 | -87.32 | 15.73 |
| Angular Gyrus R | 42.56 | -65.80 | 32.84 |
| Angular Gyrus L | -40.18 | -67.60 | 32.58 |
| Supramarginal Gyrus R | 48.28 | -49.33 | 33.37 |
| Supramarginal Gyrus L | -48.98 | -51.56 | 30.19 |
| Cingulate Gyrus R | 8.54 | -14.98 | 37.32 |
| Cingulate Gyrus L | -8.43 | -16.72 | 37.14 |
| Inferior Parietal Lobule R | 47.65 | -42.97 | 39.86 |
| Inferior Parietal Lobule L | -46.17 | -43.19 | 40.42 |
| Precuneus R | 16.83 | -65.32 | 42.40 |
| Precuneus L | -16.11 | -65.22 | 42.33 |
| Superior Parietal Lobule R | 27.28 | -61.83 | 56.16 |
| Superior Parietal Lobule L | -24.40 | -62.55 | 55.63 |
| Middle Frontal Gyrus R | 33.82 | 27.48 | 25.58 |
| Middle Frontal Gyrus L | -33.04 | 27.25 | 27.59 |
| Paracentral Lobule R | 8.73 | -36.21 | 60.96 |
| Paracentral Lobule L | -9.90 | -34.07 | 58.52 |
| Postcentral Gyrus R | 39.09 | -31.93 | 50.06 |
| Postcentral Gyrus L | -40.81 | -29.56 | 47.47 |
| Precentral Gyrus R | 43.96 | -11.88 | 40.57 |
| Precentral Gyrus L | -40.40 | -14.17 | 45.59 |
| Superior Frontal Gyrus R | 18.69 | 37.04 | 32.18 |
| Superior Frontal Gyrus L | -16.05 | 39.52 | 28.41 |
| Medial Frontal Gyrus R | 10.70 | 28.26 | 28.05 |
| Medial Frontal Gyrus L | -8.28 | 22.81 | 35.31 |

# References

1. Baker AP, et al. (2014) Fast transient networks in spontaneous human brain activity*. Eli*fe 3. doi:10.7554/eLife.01867.
